# Socio-cultural integration of Afghan refugees in Türkiye: the role of traumatic events, post-displacement stressors and mental health

**DOI:** 10.1017/S204579602300063X

**Published:** 2023-08-04

**Authors:** Gülşah Kurt, Maryam Ekhtiari, Peter Ventevogel, Merve Ersahin, Zeynep Ilkkursun, Nuriye Akbiyik, Ceren Acarturk

**Affiliations:** 1 Discipline of Psychiatry and Mental Health, UNSW, Sydney, Australia; 2 Department of Sociology, Koc University, Istanbul, Turkey; 3 Public Health Section, United Nations High Commissioner for Refugees, Geneva, Switzerland; 4 Department of Clinical Psychology, Erasmus University, Rotterdam, The Netherlands; 5 Department of Psychology, Koc University, Istanbul, Turkey; 6 Faculty of Humanities and Social Sciences, University of Bergamo, Bergamo, Italy

**Keywords:** Afghan refugees, mental health, resources, socio-cultural integration, socioecological context, Türkiye

## Abstract

**Aims:**

Socio-cultural integration of refugees has received scant attention in the academic literature. Türkiye hosts the largest number of refugees, including Afghans, as the second largest asylum-seeking group in Türkiye. There is a dearth of research into the mental health and integration of Afghan refugees in Türkiye. The aim of the present study was to investigate socio-cultural integration outcomes among Afghan refugees in Türkiye by considering the role of traumatic events and post-displacement stressors. The role of mental health in integration outcomes was further examined.

**Methods:**

A cross-sectional, web-based survey study with 785 Afghan refugees in Türkiye was conducted between April and June 2021. Data were collected on socio-demographic characteristics, potentially traumatic events (PTEs) (Harvard Trauma Questionnaire), post-displacement stressors (Post-Migration Living Difficulties Checklist), mental health symptoms (Hopkins Symptoms Checklist-25), social integration- *social bonds* (contact with co-ethnic group members) and *social bridges* (contact with the host community)- and cultural integration (Vancouver Index of Acculturation). Structural equation modelling was conducted to test the hypothesized relationship between conflict and displacement-related stressors, mental health and socio-cultural integration.

**Results:**

Findings showed that conflict-related traumatic events and post-displacement stressors significantly predicted higher mental health symptoms. Experiencing traumatic events significantly predicted higher levels of social bridges, adopting destination culture and lower level of maintaining heritage culture. Mental health problems predicted the relationship between stressors related to forced displacement and integration outcomes-social bridges and adopting destination culture. These findings highlight the role of mental health as an indispensable resource for socio-cultural integration. Further, conflict and displacement-related stressors are important determinants of socio-cultural integration among Afghan refugees in Türkiye.

**Conclusion:**

Exposure to PTEs and post-displacement stressors were significant risk factors for the mental health and socio-cultural integration of Afghan refugees in Türkiye. These stressful experiences deteriorate refugees’ mental health, which hinders their integration into the host society.

## Introduction

Global displacement hit an all-time record, with 35 million people forced to leave their countries and seek refuge in other countries (United Nations High Commissioner for Refugees, 2023). Following Syria and Ukraine, Afghanistan is among the top three countries where most refugees originate. As a torn-apart country by foreign and local forces, Afghanistan has an intricate story of displacement over more than 40 years. After the religious forces seized power in 2021, the humanitarian crisis was further accelerated, putting millions of people in need of humanitarian assistance (United Nations High Commissioner for Refugees Refugee Statistics, 2023). Türkiye is one of the main destinations for Afghans, who represent the second-largest group seeking asylum, followed by Syrians (Presidency of Migration Management, 2023).

Refugees are at higher risk of poor mental health and functioning due to traumatic experiences and stressors before, during and after migration (Patanè *et al.*, 2022). A recent meta-analysis revealed that the prevalence of depression, anxiety and post-traumatic stress disorder among refugees and asylum seekers is 31.5%, 31.4% and 11%, respectively (Blackmore *et al.*, 2020). Similar prevalence rates were also reported among Afghan refugees and asylum seekers living in neighbouring and high-income countries (Gerritsen *et al.*, 2006; Hosseini Divkolaye and Burkle, 2017; Slewa-Younan *et al.*, 2017; Walther *et al.*, 2020). Resource-based models of refugee adaptation (Ryan *et al.*, 2008) consider mental health an indispensable resource facilitating participation and successful integration. Therefore, mental health problems might hinder refugees’ social and cognitive capacities and skills necessary during the integration process (Nickerson *et al.*, 2019; Schick *et al.*, 2016). Socio-cultural integration, one of the dimensions of integration, is the combination of social capital that refugee communities maintain or develop after the resettlement and cultural adaptation (Pennix, 2005). According to the Social Capital Theory (Putnam, 1993), social relationships provide crucial capital to people whereby they can obtain resources, access emotional and instrumental support and maintain healthy functioning. Both social bonds (contact with own ethnic group) and social bridges (contact with host community members) are necessary for successful adaptation (Ager and Strang, 2008). On the other hand, cultural adaptation is often investigated during the acculturation process, which changes people’s values, behaviours and identities when they encounter a new culture. During intercultural contact, people might prefer maintaining their heritage culture and adopting the host community culture to a different degree (Berry, 1997). Both social capital and cultural adaptation are conducive to mental health and functioning among refugee communities (Chen *et al.*, 2017; Niemi *et al.*, 2019; Tartakovsky and Saranga, 2022). However, conflict-related traumatic experiences and post-displacement stressors can pose a significant threat to acquiring or maintaining relationships and successfully integrating into the host community (Kurt *et al.*, 2021) (Tartakovsky and Saranga, 2022). So far, existing studies have predominantly focused on economic integration among refugees in high-income countries (Bakker *et al.*, 2014; Stempel and Alemi, 2021; Walther *et al.*, 2020). There is a dearth of studies examining the sociocultural integration among refugees in low- and middle-income countries where the majority live in. Further, evidence on the mental health and integration of Afghan refugees is extremely limited, albeit much needed in a given socio-political atmosphere.

The present study aimed to investigate the socio-cultural integration among Afghan refugees in Türkiye by considering the role of conflict and displacement stressors. Building on the resource-based model of refugee adaptation (Ryan *et al.*, 2008), we examined the role of mental health in explaining the relationship between those stressors and socio-cultural integration. We posited that traumatic events and post-displacement stressors would be positively associated with mental health symptoms and negatively with socio-cultural integration outcomes. Mental health symptoms, then, would predict lower socio-cultural integration outcomes.

## Method

### Procedure

**Figure 1.**
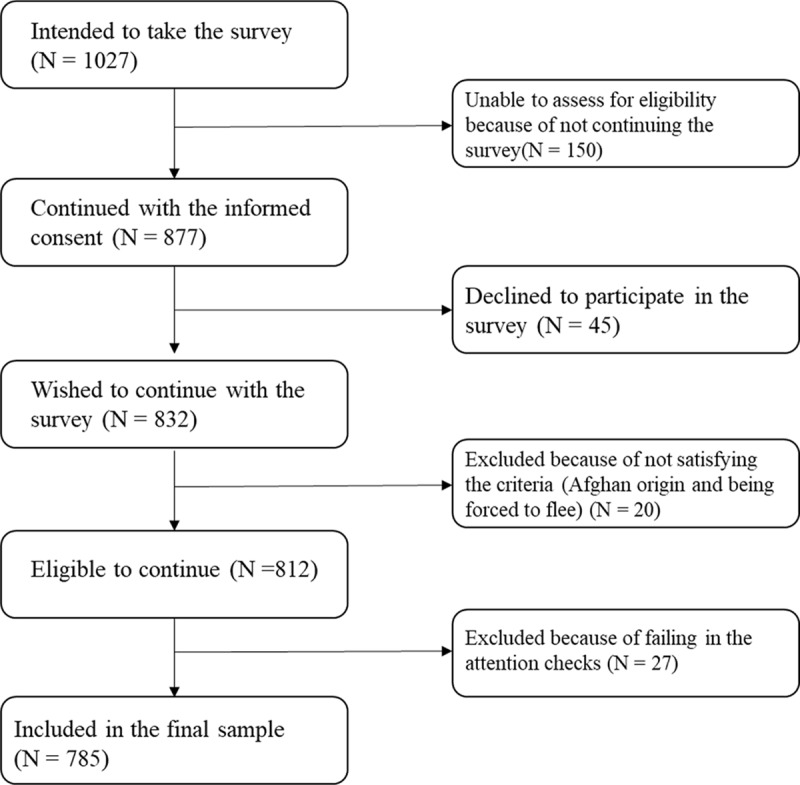
Participant flow.

A cross-sectional, web-based survey study [2020.423.IRB3.161] was conducted between April and June 2021 (Kurt *et al.*, 2022). Participants were recruited through a non-governmental organization providing services to Afghan refugees in Türkiye. The online survey link was shared via the social media platforms of this organization. The study inclusion criteria were (1) being above 18 years, (2) being literate in the Dari language, (3) having Afghan origin and (4) fled to Türkiye due to political and economic unrest in Afghanistan. Two screening questions (Are you forcibly displaced due to political and economic unrest in Afghanistan? and Are you coming from Afghan origin?) were asked at the beginning of the survey. Those who said yes to both were allowed to proceed with the rest of the survey. Flow chart of the participants’ recruitment is given in Fig. 1.

Completion of the survey lasted approximately half an hour. To compensate for time and effort of the participants, they were asked to provide their contact information (either their email addresses or phone numbers) at the end of the survey. Research assistants contacted participants and sent the digital grocery check of 3.75 US dollars.

### Measures

#### Potentially traumatic events

Part one of the Harvard Trauma Questionnaire (Mollica *et al.*, 1992) was used to measure the potentially traumatic events (PTEs) that the participants have experienced before, during and or after migrating to Türkiye. The instrument includes 17 items (e.g., “lack of food or water,” “combat situation,” “imprisonment”), each rated on a 0 (no – absent) to 1 (yes – present) scale. Sum scores are calculated as ranging from 0 to 17. Higher scores indicate a higher number of PTEs experienced by the participants. We used the validated Dari version (Wind *et al.*, 2017). As the items are answered on a categorical scale, internal consistency was evaluated by calculating Omega, which was 0.82 in the present study.

#### Post-displacement stressors

Post-Migration Living Difficulties Checklist (Schick *et al.*, 2016; Silove *et al.*, 1997) is a 17-item scale that measures the wide range of challenges such as social, economic and legal stressors that participants have been experiencing in the last 12 months. Items are rated on a 5-point Likert Scale, ranging from 0 (not a problem) to 4 (very serious problem). Higher scores indicate a higher level of post-displacement stressors experienced in the last 12 months. The scale was translated and back translated by bilingual researchers in the present study. Any inconsistencies between the original and back-translated document were resolved by relying on the original version. The Cronbach’s alpha for this study was 0.89.

#### Mental health symptoms

Mental health symptoms as an indicator of psychological resources were evaluated using Hopkins Symptom Checklist (Mollica *et al.*, 1987) with 25 items focusing on symptoms of anxiety and depression. Participants are asked to indicate how much they are bothered or distressed by the given symptoms (e.g., feeling fearful and trembling for anxiety symptoms and feeling low in energy, slowed down and feeling lonely for depressive symptoms) in the last week, including today. Each item is rated on a 4-point Likert Scale (1 = not at all, 4 = extremely), which higher scores indicate a higher level of mental health symptoms. The scale was previously validated with displaced Afghans (Wind *et al.*, 2017). The Cronbach’s alpha for this study was 0.92 for anxiety and 0.94 for depression. We used the overall mean score of the scale as an indicator of mental health symptoms.

#### Integration outcomes

##### Social integration

Following the conceptualization by Ager and Strang (2008) on different domains of integration, we focused on assessing *social bonds* (contact with co-ethnic group members) and *social bridges* (contact with the host community) as the indicators of social integration. We used four items to measure quantity (frequency of contact) and quality of contact (valence of contact) with their own ethnic group and host community members (Barlow *et al.*, 2012). Social bonds consist of the following items: How frequently do you contact people from your ethnic group? and How frequently do you have positive contact with people from your ethnic group? Social bridges consist of the following items: How frequently do you contact Turkish people? and How frequently do you have positive contact with Turkish people? Items were translated and back-translated by bilingual researchers. We combined the items for quality and quantity of contact with own ethnic group as *social bonds* and with Turkish people as *social bridges*. The Cronbach’s alpha was 0.84 for social bonds and 0.76 for social bridges in the present study.

##### Cultural integration

As indicators of cultural integration, acculturation orientations as maintenance of heritage culture and adoption of destination culture were measured by the Vancouver Index of Acculturation (Ryder *et al.*, 2000). Participants are asked to rate on a 9-point Likert scale (1 = strongly disagree, 9 = strongly agree) to what extent they agree to maintain the values and norms of their heritage culture and embrace the culture of the destination country in different domains of life such as private and public domains. It has 20 items mirroring each other, with the only difference about reference to the heritage or destination culture. Example items are “I often participate in my heritage cultural traditions” (maintenance of heritage culture) and “I often participate in mainstream cultural traditions” (adoption of destination culture). Ten items referring to heritage culture are averaged to the maintenance of heritage culture, and 10 items referring to destination culture (Turkish culture) constitute the adoption of destination culture. Items were translated and back-translated by bilingual researchers. The scale has often been used for culturally diverse communities to understand their integration process in the resettlement context (Testa *et al.*, 2019; Zhang *et al.*, 2010). Higher scores indicate greater endorsement of the respective culture’s values, norms and practices. The Cronbach’s alpha for heritage culture maintenance was 0.83, and 0.81 for adopting destination culture in the present study.

### Data analysis

Descriptive and bivariate correlations were performed using SPSS Statistics 28.0 (IBM Corp, 2021). Confirmatory factor analysis (CFA) and structural equation modelling were conducted to estimate the parameters for the hypothesized model with Mplus Software 8 (Muthén and Muthén, 1998-2003). To prevent sample size reduction and subsequent loss of power, full information maximum likelihood estimation was employed to handle missing data at random (Enders, 2010).

We used a two-step approach in our analysis. As we used best proxy indicators for social and cultural integration outcome, we conducted a CFA in the first step to test whether the indicators adequately reflected the proposed latent factors for cultural and social integration constructs. In our model, we included cultural and social integration as latent variables and PTEs, post-displacement stressors and mental health symptoms as manifest variables. In the second step, we tested the structural equation model by using top-down model building where we included all the relevant covariates (age, sex, education, marital status, income, legal status and length of stay) and then removed non-significant paths one at a time to preserve model parsimony (Wang and Wang, 2012). The significance of indirect predictor role of traumatic events and post-displacement stressors on integration outcomes via mental health symptoms was tested using the bootstrapping technique (1000 resampling) with 95% confidence intervals (MacKinnon *et al.*, 2007). Standardized coefficients were presented in the final model. Following model fit indices were used to evaluate the overall goodness of model fit: chi-square goodness of fit test, comparative fit index (CFI) >.90, root mean square error of approximation (RMSEA) <.08 with 90% CI and standardized root mean square residual (SRMR) <.08 (Brown, 2006; Kline, 2011; MacCallum *et al.*, 1996).

## Results

### Participants’ characteristics

The current sample consisted of 785 Afghans (33.8% female). The mean age of the participants was 29.60 years (SD, 9.50 years), ranging from 18 to 67 years. Among the participants, half were married (49.9%), had at least one child (50.3%) and were asylum seekers (55.2%). The mean duration of stay in Türkiye was 36.32 months (SD, 24.43 months), and the mean number of years of education was 11.35 years (SD, 4.24 years). Most participants’ monthly household income (80%) was under 300 US dollars. Demographic characteristics of the current sample (e.g., age, gender, education, legal status) are similar to those reported in a large-scale national survey in Türkiye (Eryurt and Koc, 2017).

The most frequently reported PTEs were “living in a combat situation (66.4%)” and “lack of food or water (56.1%)” while “brainwashing (5.4%)” and “rape or sexual abuse (6.6)” were the least reported incidents. The details are given in Table 1.

**Table 1.** Sample characteristics (*n* = 785)

Gender (female), *n* (%)	265 (33.8)
Age, Mean (SD)	29.60 (9.50)
Marital status, *n* (%)	
Married	392 (49.9)
Unmarried	349 (44.5)
Divorced	27 (3.4)
Widowed	17 (2.2)
Children (yes), *n* (%)	395 (50.3)
Income, *n* (%)	
<1000 Turkish liras	201 (25.6)
1001−2000 Turkish liras	294 (37.5)
2001−5000 Turkish liras	139 (17.7)
Financial aid (yes), *n* (%)	120 (15.3)
Legal status, *n* (%)	
Asylum seeker	433 (55.2)
Conditional refugee	71 (9)
Residency permit	138 (17.6)
Turkish nationality	9 (1.1)
No legal status	134 (17)
Education level in years, Mean (SD)	11.35 (4.24)
Length of stay in months, Mean (SD)	36.32 (24.43)
Number of experiencing potentially traumatic incidents, Mean (SD)	5.56 (3.70)
Post migration living difficulties, Mean (SD)	2.12 (.83)

### Descriptive statistics

Table 2 presents the descriptive statistics and bivariate correlations. Female gender and older age were negatively correlated with social bridges and adoption of the destination culture and positively correlated with mental health symptoms. Higher education and income level were positively correlated with social bridges, and higher income was positively correlated with social bonds. Income level was also negatively correlated with mental health symptoms. Compared to holding a legal status, asylum-seeking was negatively correlated with social bridges and positively correlated with mental health symptoms. Having no legal status was negatively associated with the adoption of the destination culture compared to holding a legal status.

**Table 2.** Descriptive statistics and bivariate correlations among study variables

Variable	1	2	3	4	5	6	7	8	9	10	11	12	13	14	15
1. Social bridges	–														
2. Social bonds	.237**	–													
3. Maintaining heritage culture	.040	.348**	–												
4. Adopting to host culture	.491**	.057	.232**	–											
5. Mental health symptoms	−.182**	−.093*	−.098*	−.193**	–										
6. Post-displacement stressors	−.074	−.094*	−.053	−.068	.600**	–									
7. Traumatic experiences	.068	−.059	−.124**	.013	.499**	.553**	–								
8. Age	−.169**	−.025	.011	−.107**	.104**	−.043	.014	–							
9. Income	.224**	.084*	.061	.071	−.118*	−.018	−.024	−.076	–						
10. Education	.155**	.011	.068	.151**	−.045	.078*	.064	−.115**	.082*	–					
11. Length of stay	.013	.004	.003	−.004	−.026	−.041	.007	−.032	.077	−.014	–				
12. Sex (0 = male, 1 = female)	−.242**	.002	.007	−.186**	.238**	.002	−.080*	.099**	−.255**	−.185**	−.032	–			
13. Marital status (0 = not married, 1 = married)	−.097*	.010	.023	−.056	−.002	−.055	−.050	.441**	.012	−.048	−.001	.133**	–		
14. Legal status 1 (legal status vs. asylum seeking)	−.085*	−.045	−.076	−.072	.123**	.050	.052	−.001	−.126**	−.039	−.013	.067	.004	–	
15. Legal status 2 (legal status vs. non-legal status)	.076	−.023	.057	−.109**	−.051	.178**	.127**	−.111**	.160**	.007	.005	−.162**	−.094**	−.503**	–
*N*	650	650	653	653	687	712	785	769	641	766					
Mean	3.53	3.05	5.29	5.36	2.19	2.11	5.56	29.60	3.36	11.35					
SD	1.00	1.03	.83	.79	.73	.83	3.70	9.50	1.51	4.24					
Range	1−5	1−5	2−7	2−7	1−4	0−4	0−17	18−67	1−10	0−21					
Skewness	−.362	−.089	−.632	−.559	.285	−.414	.481	1.52	.207	−1.059					
Kurtosis	−.446	−.476	.132	.146	−.865	−.367	−.383	2.326	.518	1.020					

* *p* < 0.05, **p < 0.01, ***p < 0.001.

### Measurement model testing

CFA results indicated an overall good model fit for both social and cultural integration outcomes. The results supported our initial conceptualization of two dimensions of social integration, namely social bonds and bridges. Allowing two factors to covary (Hayduk and Littvay, 2012), the model was just identified with factor loadings ranging from 0.75 to 0.95, indicating acceptable loadings. Further, the original two factors structure of cultural integration (maintenance of heritage culture and adoption of destination culture) were supported. The final model for this measure fit the data well (χ^2^(168) = 469.776, *p* < 0.001, CFI = 0.911, RMSEA = 0.052 (90% CI = 0.047-0.058), SRMR = 0.053) with factors loading ranging from 0.49 to 0.73, indicating acceptable loading values.

### Structural equation model testing

The model fit the data well, (χ^2^(356) = 829.104, *p* < 0.001, CFI = 0.908, RMSEA = 0.042 (90% CI = 0.038-0.046), SRMR = 0.052). PTEs (*β* = 0.267, *p* < 0.001) and post-displacement stressors (*β* = 0.452 *p* < 0.001) positively predicted mental health symptoms. There was a negative association between PTEs and maintenance of heritage culture (*β* = −0.119 *p* < 0.05), but positive association with adoption of destination culture (*β* = 0.127 *p* < 0.05) and social bridges (*β* = 0.259, *p* < 0.001). Mental health symptoms negatively predicted social bridges and adoption of destination culture (*β* = −0.342, *p* < 0.001, *β* = −0.266, *p* < 0.001, respectively).

The relation of PTEs and post-displacement stressors on adoption of destination culture via mental health symptoms were significant (*β* = −0.071, 95% CI: [−0.112 to −0.038], *β* = −0.120, 95% CI: [−0.175 to −0.067], respectively). That is, in our model, traumatic events and displacement stressors positively predicted a higher level of mental health symptoms, negatively predicting the adoption of the destination culture. Further, mental health symptoms predicted the relationship of traumatic events with social bridges (*β* = −0.091, 95% CI: [−0.136 to −0.055]) and of post-displacement stressors with social bridges (*β* = −0.155, 95% CI: [−0.208 to −0.097]). Similar to the adoption of destination culture, traumatic events and post-displacement stressors were associated with higher mental health symptoms, predicting a lower level of adoption of destination culture. Significant paths are depicted in Fig. 1, and Table 3 includes the direct and indirect predictions the tested model.

**Table 3.** Direct and indirect effects

		95% CI^a^	
	*β* (SE)	LLCI	ULCI
***Direct Paths***			
TE → MS	.267*** (.039)	.189	.339
PMLD → MS	.452*** (.035)	.384	.522
TE → MHC	−.119* (.055)	−.225	−.005
TE → ADC	.127* (.059)	.015	.241
PMLD → MHC	.057 (.057)	−.065	.162
PMLD → ADC	.001 (.065)	−.127	.123
TE → SBridge	.259*** (.058)	.135	.372
TE → SBond	.014 (.058)	−.100	.129
PMLD → SBridge	−.027 (.066)	−.151	.100
PMLD → SBond	−.059 (.060)	−.179	.052
MS → MHC	−.081 (.057)	−.186	.031
MS → ADC	−.266*** (.058)	−.378	−.150
MS → SBridge	−.342*** (.057)	−.450	−.228
MS → SBond	−.093 (.057)	−.209	.019
***Indirect Paths***			
TE→ MS → MHC	−.022 (.015)	−.051	.009
TE→ MS → ADC	−.071*** (.019)	−.112	−.038
TE→ MS → SBridge	−.091*** (.021)	−.136	−.055
TE→ MS → SBond	−.025 (.016)	−.059	.005
PMLD→ MS → MHC	−.037 (.026)	−.087	.014
PMLD → MS → ADC	−.120*** (.028)	−.175	−.067
PMLD → MS → SBridge	−.155*** (.028)	−.208	−.097
PMLD → MS → SBond	−.042 (.026)	−.094	.008

* *p* < 0.05; **p < 0.01; ***p < 0.001. TE: Traumatic experiences, PMLD: Post-displacement stressors, MS: Mental health symptoms, MHC: Maintenance of the heritage culture, ADC: Adoption of the destination culture, Sbridge: Social bridges, Sbond: Social bonds.

a Confidence intervals of standardized results are reported. LLCI: lower level confidence interval. ULCI: upper level confidence interval.

Given the cross-sectional nature of the present study, the alternative model where mental health was the outcome variable and social and cultural integration were the intervening variables was tested. The model did not fit the data well, CFI = 0.847, RMSEA = 0.054, 90% CI 0.050–0.057, SRMR = 0.097. Therefore, the initially proposed model was supported and explained in the following section.

## Discussion

We found that traumatic events and post-displacement stressors significantly predicted higher mental health symptoms among Afghan participants. Traumatic events also predicted a higher level of social bridges and adoption of Turkish culture but a lower level of maintenance of Afghan culture. Mental health played a significant role in the relationship between traumatic experiences, post-displacement stressors and social bridges and adoption.

Compared to the large body of research on refugee mental health, evidence on the refugee integration process is sparse, especially among those resettled in low- and middle-income countries. Therefore, our findings provide novel insights into the socio-cultural integration processes of refugees in those countries. This is the first study in a low- and middle- income country to explore the relationship between mental health and integration, considering the role of conflict and displacement-related stressors. Unlike the studies conducted in high-income income countries (Kartal *et al.*, 2018; Tartakovsky and Saranga, 2022), we found that experiencing traumatic incidents do not necessarily hamper the capacity of refugees to develop a relationship with host community members. Yet, consistent with the previous findings (Kurt *et al.*, 2021), they are likely to hinder maintaining heritage culture. These findings might be explained by the notion that stressful life events mobilize various coping strategies to help deal with associated psychological distress, ultimately determining the aftermath of PTEs (Lazarus and Folkman, 1984). Meta-analytic evidence attests that flexibility in using different coping strategies provides an advantage in terms of psychological adjustment after stressful life events (Cheng *et al.*, 2014). As such, those who experienced a higher level of traumatic events might have chosen to integrate into the host society as a coping strategy to deal with negative feelings related to traumatic events. Concurrently, they might have engaged in avoidance coping strategy and eschewed their heritage culture as it is likely to reinvigorate those excruciating traumatic memories. After experiencing traumatic events related to group membership, individuals might choose to maintain or disregard their group identity, depending on whether their group provides resources to combat stressors (Muldoon *et al*., 2019). While maintaining heritage culture may not help Afghans to cope with stressors, adopting Turkish culture might provide the necessary resources to adjust to a new life. On the other hand, post-displacement stressors were not associated with any of the integration outcomes. Conflict-related traumatic events seem to play a major role in shaping the integration process, while post-displacement stressors are a more potent risk factor for mental health (Hou *et al.*, 2020).

Our findings highlight the importance of mental health as an important asset for integration, requiring forming new relationships and learning about a new culture. Mental health problems might hinder the attainment of new resources, such as learning a new language and navigating in a new setting which is essential for integrating into the host society (Ryan *et al.*, 2008). However, to maintain social ties with ethnic group members or preserve heritage culture, such resources requiring energy and skills may not be necessary. Thus, it is essential to provide mental health support to foster the integration of refugees in a new resettlement context. A growing body of evidence shows that cultural-adapted brief psychosocial interventions such as Self-Help Plus (SH+) and Problem-Management Plus (PM+) might be effective in preventing and treating mental health problems among refugees (Acarturk et al., 2022; Alemi et al, 2023). As most Afghans lack opportunities to use healthcare services due to their legal status in Türkiye (Eryurt and Koç, 2017), future initiatives to provide such psychosocial interventions are urgently needed.

Our findings on socio-demographic characteristics are important to show at-risk groups to guide the efforts of policymakers and practitioners. Similar to previous research, female gender, older age, lower education and income level were found to be associated with worse mental health and integration outcomes (Alemi *et al.*, 2014). Compared to holding a legal status, asylum-seeking or having no legal status was associated with mental health problems and integration difficulties in our sample. Uncertainty around the asylum-seeking process has prolonged adverse impacts on the psychological adjustment of refugees because of compounded living difficulties such as unstable housing and employment (Nickerson *et al.*, 2011). This finding highlights the importance of stable residency status for those fleeing from violence and persecution as it provides access to basic services that are conducive to mental health and integration (Posselt *et al.*, 2019; Li *et al.*, 2016; Steel *et al.*, 2011).

The present study has some limitations. First, the cross-sectional design of the study does not allow us to make any causal inferences about the relationship between the study variables. Although our hypothesized model is based on theoretical considerations and the alternative model was tested, the directions of the relationships might change. Thus, longitudinal studies investigating the hypothesized relationship are warranted. Second, online data collection might have biased our sample by recruiting only those with access to the internet and technological devices. Yet, online tools became one of the most viable options to expand the reach of surveys, especially following COVID-19 (Dron *et al.*, 2022). The similarity of our sample characteristics to the large-scale, nationwide study of Afghans in Türkiye supports the representativeness of our sample, hence the potential of our results for generalizability.

Further, we only focused on the socio-cultural dimension of integration. In order to provide a complete picture of integration, a comprehensive investigation including other dimensions such as economic and political is required. Lastly, the present study only included the perspective of refugees. However, integration is a two-way process (Ager & Strang, 2008). Host community members’ attitudes and preferences are important determinants of integration (Esses *et al.*, 2017). Therefore, future studies might consider investigating the impact of perceived or actual preferences of the host community on refugee integration.

## Data Availability

The data that support the findings of this study are available from the corresponding author (C.A.) on reasonable request.

## References

[ref1] Acarturk C, Uygun E, Ilkkursun Z, Carswell K, Tedeschi F, Batu M, Eskici S, Kurt G, Anttila M, Au T, Baumgartner J, Churchill R, Cuijpers P, Becker T, Koesters M, Lantta T, Nosè M, Ostuzzi G, Popa M, Purgato M, Sijbrandij M, Turrini G, Välimäki M, Walker L, Wancata J, Zanini E, White RG, van Ommeren M and Barbui C (2022) Effectiveness of a WHO self-help psychological intervention for preventing mental disorders among Syrian refugees in Turkey: A randomized controlled trial. *World Psychiatry* 21, 88–95. 35015365 10.1002/wps.20939 PMC8751562

[ref2] Ager A and Strang A (2008) Understanding integration: A conceptual framework. *Journal of Refugee Studies* 21, 166–191.

[ref3] Alemi Q, Panter-Brick C, Oriya S, Ahmady M, Alimi AQ, Faiz H, Hakim N, Hashemi SAS, Manaly MA, Naseri R and Parwiz K (2023). Afghan mental health and psychosocial well-being: thematic review of four decades of research and interventions. *BJPsych open*, 9(4), e125. 10.1192/bjo.2023.502 PMC10375890 37424447

[ref4] Alemi Q, James S, Cruz R, Zepeda V and Racadio M (2014) Psychological distress in afghan refugees: A mixed-method systematic review. *Journal of Immigrant and Minority Health* 16, 1247–1261. 23784146 10.1007/s10903-013-9861-1 PMC3912229

[ref5] Bakker L, Dagevos J and Engbersen G (2014) The importance of resources and security in the socio-economic integration of refugees. A study on the impact of length of stay in asylum accommodation and residence status on socio-economic integration for the four largest refugee groups in the Netherlands. *Journal of International Migration and Integration* 15, 431–448.

[ref6] Barlow FK, Paolini S, Pedersen A, Hornsey MJ, Radke HRM, Harwood J, Rubin M and Sibley CG (2012) The contact caveat: Negative contact predicts increased prejudice more than positive contact predicts reduced prejudice. *Personality & Social Psychology Bulletin* 38, 1629–1643. 22941796 10.1177/0146167212457953

[ref7] Berry JW (1997) Lead article - immigration, acculturation, and adaptation. *Applied Psychology* 46, 5–34.

[ref8] Blackmore R, Boyle JA, Fazel M, Ranasinha S, Gray KM, Fitzgerald G, Misso M and Gibson-Helm M (2020) The prevalence of mental illness in refugees and asylum seekers: A systematic review and meta-analysis. *PLoS Medicine* 17, e1003337. 10.1371/journal.pmed.1003337 PMC7505461 32956381

[ref9] Brown TA (2006) *Confirmatory Factor Analysis for Applied Research*. New York: Guilford Press.

[ref10] Cheng C, Lau HPB and Chan MPS (2014) Coping flexibility and psychological adjustment to stressful life changes: A meta-analytic review. *Psychological Bulletin* 140, 1582–1607. 25222637 10.1037/a0037913

[ref11] Chen W, Ling L and Renzaho AMN (2017) Building a new life in Australia: An analysis of the first wave of the longitudinal study of humanitarian migrants in Australia to assess the association between social integration and self-rated health. *BMJ Open* 7, e014313. 10.1136/bmjopen-2016-014313 PMC5353341 28298368

[ref12] Dron L, Kalatharan V, Gupta A, Haggstrom J, Zariffa N, Morris AD, Arora P and Park J (2022) Data capture and sharing in the COVID-19 pandemic: A cause for concern. *The Lancet Digital Health* 4, e748–e756. 36150783 10.1016/S2589-7500(22)00147-9 PMC9489064

[ref13] Enders CK (2010) *Applied Missing Data Anlysis*. New York: Guilford Press.

[ref14] Eryurt MA and Koç İ (2017) Türkiye’de Afganistan Uyruklu Uluslararası Koruma Başvurusu ve Statüsü Sahipleri Üzerine Analiz: Türkiye’ye geliş sebepleri, Türkiye’de Kalışları, Gelelecek Planları ve Amaçları. Ankara: Hacettepe University Press.

[ref15] Esses VM, Hamilton LK and Gaucher D (2017) The global refugee Crisis: Empirical evidence and policy implications for improving public attitudes and facilitating refugee resettlement. *Social Issues and Policy Review* 11, 78–123.

[ref16] Gerritsen AAM, Bramsen I, Devillé W, van Willigen LHM, Hovens JE and van der Ploeg HM (2006) Physical and mental health of Afghan, Iranian and Somali asylum seekers and refugees living in the Netherlands. *Social Psychiatry and Psychiatric Epidemiology* 41, 18–26. 16341619 10.1007/s00127-005-0003-5

[ref17] Hayduk LA and Littvay L (2012) Should researchers use single indicators, best indicators, or multiple indicators in structural equation models? *BMC Medical Research Methodology* 12, 159. 10.1186/1471-2288-12-159 PMC3506474 23088287

[ref18] Hosseini DNS and Burkle FM (2017) The enduring health challenges of Afghan immigrants and refugees in Iran: A systematic review. *PLoS Currents* 9, 1–13. 10.1371/currents.dis.449b4c549951e359363a90a7f4cf8fc4 PMC5554007 28856065

[ref19] Hou WK, Liu H, Liang L, Ho J, Kim H, Seong E, Bonanno GA, Hobfoll SE and Hall BJ (2020) Everyday life experiences and mental health among conflict-affected forced migrants: A meta-analysis. *Journal of Affective Disorders* 264, 50–68. 31846902 10.1016/j.jad.2019.11.165 PMC7064367

[ref20] IBM Corp (2021) IBM SPSS Statistics for Windows, Version 28.0.

[ref21] Kartal D, Alkemade N, Eisenbruch M and Kissane D (2018) Traumatic exposure, acculturative stress and cultural orientation: The influence on PTSD, depressive and anxiety symptoms among refugees. *Social Psychiatry and Psychiatric Epidemiology* 53, 931–941. 29931441 10.1007/s00127-018-1532-z

[ref22] Kline RB (2011) *Principles and Practice of Structural Equation Modeling*, 3rd edn. London: Guilford Press The Guilford Press, New York.

[ref23] Kurt G, Acar İH, Ilkkursun Z, Yurtbakan T, Acar B, Uygun E and Acarturk C (2021) Traumatic experiences, acculturation, and psychological distress among Syrian refugees in Turkey: The mediating role of coping strategies. *International Journal of Intercultural Relations* 81, 214–225.

[ref24] Kurt G, Ventevogel P, Ekhtiari M, Ilkkursun Z, Ersahin M, Akbiyik N and Acarturk C (2022) Estimated prevalence rates and risk factors for common mental health problems among Syrian and Afghan refugees in Türkiye. *BJPsych Open* 8(5), e167. 10.1192/bjo.2022.573 PMC9534906 36106400

[ref25] Lazarus RS and Folkman S (1984) The stress concept in the life sciences. In Lazarus Richard and Folkman Susan (eds.), *Stress, Appraisal, and Coping*. New York: Springer, 141–144.

[ref26] Li SSY, Liddell BJ, Nickerson A (2016) The relationship between post-migration stress and psychological disorders in refugees and asylum seekers. *Current Psychiatry Reports* 18, 1–9. 27436307 10.1007/s11920-016-0723-0

[ref27] MacCallum RC, Browne MW and Sugawara HM (1996) Power analysis and determination of sample size for covariance structure modeling. *Psychological Methods* 1, 130–149.

[ref28] MacKinnon DP, Fairchild AJ and Fritz MS (2007) Mediation analysis. *Annual Review of Psychology* 58, 593–614. 10.1146/annurev.psych.58.110405.085542 PMC2819368 16968208

[ref29] Mollica RF, Caspi-Yavin Y, Bollini P, Truong T, Tor S and Lavelle J (1992) The Harvard Trauma Questionnaire: Validating a cross-cultural instrument for measuring torture, trauma, and posttraumatic stress disorder in Indochinese refugees. *Journal of Nervous and Mental Disease* 180, 111–116. 1737972

[ref30] Mollica R, Wyshak G, Marneffe D, Khuon F and Lavelle J (1987) Indochinese versions of the Hopkins Symptom Checklist-25: A screening instrument for the psychiatric care of refugees. *American Journal of Psychiatry* 144, 497–500. 3565621 10.1176/ajp.144.4.497

[ref31] Muldoon OT, Haslam SA, Haslam C, Cruwys T, Kearns M and Jetten J (2019) The social psychology of responses to trauma: social identity pathways associated with divergent traumatic responses. *European Review of Social Psychology*, 30(1), 311–348.

[ref32] Muthén L and Muthén B (1998–2023) *Mplus User’s Guide*, 8th edn. Los Angeles, CA: Muthén & Muthén.

[ref33] Nickerson A, Liddell BJ, Keegan D, Edwards B, Felmingham KL, Forbes D, Hadzi-Pavlovic D, McFarlane AC, O’Donnell M, Silove D, Steel Z, Van Hooff M and Bryant RA (2019) Longitudinal association between trust, psychological symptoms and community engagement in resettled refugees. *Psychological Medicine* 49, 1661–1669. 30160232 10.1017/S0033291718002246

[ref34] Nickerson A, Steel Z, Bryant R, Brooks R and Silove D (2011) Change in visa status amongst Mandaean refugees: Relationship to psychological symptoms and living difficulties. *Psychiatry Research* 187, 267–274. 21296428 10.1016/j.psychres.2010.12.015

[ref35] Niemi M, Manhica H, Gunnarsson D, Ståhle G, Larsson S and Saboonchi F (2019) A scoping review and conceptual model of social participation and mental health among refugees and asylum seekers. *International Journal of Environmental Research and Public Health* 16, 4027. 10.3390/ijerph16204027 PMC6843961 31640210

[ref36] Patanè M, Ghane S, Karyotaki E, Cuijpers P, Schoonmade L, Tarsitani L and Sijbrandij M (2022) Prevalence of mental disorders in refugees and asylum seekers: A systematic review and meta-analysis. *Global Mental Health* 9, 250–263. 36618716 10.1017/gmh.2022.29 PMC9806970

[ref37] Pennix R (2005) Integration of migrants: Economic, social, cultural and political dimensions. In Miroslav M, MacDonald AL and Haug W (eds.), *The New Demographic Regime: Population Challenges and Policy Responses* Geneva: UNECE, 137–152.

[ref38] Posselt M, Eaton H, Ferguson M, Keegan D and Procter N (2019) Enablers of psychological well-being for refugees and asylum seekers living in transitional countries: A systematic review. *Health & Social Care in the Community* 27, 808–823. 30417476 10.1111/hsc.12680 PMC7380010

[ref39] Presidency of Migration Management (2023) Up-to-date Statistics. https://en.goc.gov.tr/ (accessed 29 June 2023).

[ref40] Putnam Robert (1993) The Prosperous Community: Social Capital and Public Life. *American Prospect* 13, 35–42.

[ref41] Ryan D, Dooley B and Benson C (2008) Theoretical perspectives on post-migration adaptation and psychological well-being among refugees: Towards a resource-based model. *Journal of Refugee Studies* 21, 1–18.

[ref42] Ryder AG, Alden LE and Paulhus DL (2000) Is acculturation unidimensional or bidimensional? A head-to-head comparison in the prediction of personality, self-identity, and adjustment.. *Journal of Personality and Social Psychology* 79, 49–65. 10909877 10.1037//0022-3514.79.1.49

[ref43] Schick M, Zumwald A, Knopfli B, Nickerson A, Bryant RA, Schnyder U, Muller J and Morina N (2016) Challenging future, challenging past: The relationship of social integration and psychological impairment in raumatized refugees. *European Journal of Psychotraumatology* 7(1), 28057. 10.3402/ejpt.v7.28057 PMC4756625 26886484

[ref44] Silove D, Sinnerbrink I, Field A, Manicavasagar V and Steel Z (1997) Anxiety, depression and PTSD in asylum-seekers: Associations with pre-migration trauma and post-migration stressors. *British Journal of Psychiatry* 170, 351–357. 10.1192/bjp.170.4.351 9246254

[ref45] Slewa-Younan S, Yaser A, Guajardo MGU, Mannan H, Smith CA and Mond JM (2017) The mental health and help-seeking behaviour of resettled Afghan refugees in Australia. *International Journal of Mental Health Systems* 11, 1–8. 28855961 10.1186/s13033-017-0157-z PMC5571658

[ref46] Steel Z, Momartin S, Silove D, Coello M, Aroche J and Tay KW (2011) Two year psychosocial and mental health outcomes for refugees subjected to restrictive or supportive immigration policies. *Social Science & Medicine* 72, 1149–1156. 21427011 10.1016/j.socscimed.2011.02.007

[ref47] Stempel C and Alemi Q (2021) Challenges to the economic integration of Afghan refugees in the U.S. *Journal of Ethnic and Migration Studies* 47, 4872–4892.

[ref48] Tartakovsky E and Saranga M (2022) Eritrean asylum seekers in Israel: Traumatic experience, social contacts with Eritreans and Israelis, psychological well-being, and sociocultural adaptation. *Journal of Immigrant & Refugee Studies*, 1–14.

[ref49] Testa S, Doucerain MM, Miglietta A, Jurcik T, Ryder AG and Gattino S (2019) The Vancouver Index of Acculturation (VIA): New evidence on dimensionality and measurement invariance across two cultural settings. *International Journal of Intercultural Relations* 71, 60–71.

[ref50] United Nations High Commissioner for Refugees Refugee Statistics (2023) Refugee Data Finder. https://www.unhcr.org/refugee-statistics/ (accessed 29 June 2023).

[ref51] Walther L, Kröger H, Tibubos AN, T TMT, Von Scheve C, Schupp J, Hahn E and Bajbouj M (2020) Psychological distress among refugees in Germany: A cross-sectional analysis of individual and contextual risk factors and potential consequences for integration using a nationally representative survey. *BMJ Open* 10, 1–10. 10.1136/bmjopen-2019-033658 PMC7440818 32819926

[ref52] Wang J and Wang X (2012) *Structural Equation Modeling: Applications Using Mplus*. West Sussex: John Wiley & Sons.

[ref53] Wind TR, van der AN, de la Rie S and Knipscheer J (2017) The assessment of psychopathology among traumatized refugees: Measurement invariance of the Harvard Trauma Questionnaire and the Hopkins Symptom Checklist-25 across five linguistic groups. *European Journal of Psychotraumatology* 8, 1321357. 10.1080/20008198.2017.1321357 PMC5632793 29038686

[ref54] Zhang J, Mandl H and Wang E (2010) Personality, acculturation, and psychosocial adjustment of Chinese international students in Germany. *Psychological Reports* 107, 511–525. 21117478 10.2466/07.09.11.17.PR0.107.5.511-525

